# The cisplatin-induced acute kidney injury is a novel risk factor for postoperative complications in patients with esophageal cancer: a retrospective cohort study

**DOI:** 10.1186/s12893-023-01949-0

**Published:** 2023-03-27

**Authors:** Shuhei Ueno, Miho Murashima, Ryo Ogawa, Masaki Saito, Sunao Ito, Shunsuke Hayakawa, Tomotaka Okubo, Hiroyuki Sagawa, Tatsuya Tanaka, Hiroki Takahashi, Yoichi Matsuo, Akira Mitsui, Masahiro Kimura, Takayuki Hamano, Shuji Takiguchi

**Affiliations:** 1 Department of Gastroenterological Surgery, Nagoya City University Graduate School of Medical Sciences, 1 Kawasumi, Mizuho-cho, Mizuho-ku, Nagoya, 467-8601 Japan; 2 Department of Nephrology, Nagoya City University Graduate School of Medical Sciences, 1 Kawasumi, Mizuho-cho, Mizuho-ku, Nagoya, 467-8601 Japan; 3 Department of Nephrology, Osaka University Graduate School of Medicine, 2-2 Yamadaoka, Suita, 565-0871 Japan

**Keywords:** Esophageal cancer, Acute kidney injury, Postoperative complications, Preoperative chemotherapy, Anastomotic leakage

## Abstract

**Background:**

Cisplatin-induced acute kidney injury (AKI) is common during preoperative chemotherapy for esophageal cancer. The purpose of this study was to investigate the association between AKI after preoperative chemotherapy and postoperative complications in patients with esophageal cancer.

**Methods:**

In this retrospective cohort study, we included patients who had received preoperative chemotherapy with cisplatin and underwent surgical resection for esophageal cancer under general anesthesia from January 2017 to February 2022 at an education hospital. A predictor was stage 2 or higher cisplatin-induced AKI (c-AKI) defined by the KDIGO criteria within 10 days after chemotherapy. Outcomes were postoperative complications and length of hospital stays. Associations between c-AKI and outcomes including postoperative complications and length of hospital stays were examined with logistic regression models.

**Results:**

Among 101 subjects, 22 developed c-AKI with full recovery of the estimated glomerular filtration (eGFR) before surgery. Demographics were not significantly different between patients with and without c-AKI. Patients with c-AKI had significantly longer hospital stays than those without c-AKI [mean (95% confidence interval (95%CI)) 27.6 days (23.3–31.9) and 43.8 days (26.5–61.2), respectively, mean difference (95%CI) 16.2 days (4.4–28.1)]. Those with c-AKI had higher C-reactive protein (CRP) levels and prolonged weight gain after surgery and before the events of interest despite having comparable eGFR trajectories after surgery. c-AKI was significantly associated with anastomotic leakage and postoperative pneumonia [odds ratios (95%CI) 4.14 (1.30–13.18) and 3.87 (1.35–11.0), respectively]. Propensity score adjustment and inverse probability weighing yielded similar results. Mediation analysis showed that a higher incidence of anastomotic leakage in patients with c-AKI was primarily mediated by CRP levels (mediation percentage 48%).

**Conclusion:**

c-AKI after preoperative chemotherapy in esophageal cancer patients was significantly associated with the development of postoperative complications and led to a resultant longer hospital stay. Increased vascular permeability and tissue edema due to prolonged inflammation might explain the mechanisms for the higher incidence of postoperative complications.

## Introduction

Transthoracic esophagectomy with preoperative chemotherapy is the standard treatment for advanced esophageal cancer in Japan [1]. Transthoracic esophagectomy is highly invasive, and complications, such as anastomotic leakage and postoperative pneumonia, frequently occur [2]. Risk factors for postoperative complications include older age, malnutrition, anemia, obesity, and underlying diseases such as cardiac disease and diabetes mellitus [3–5]. Postoperative complications prolong hospital stays, impair patients’ activities of daily living [6], and lead to poorer prognosis [7–9].

Preoperative chemotherapy for esophageal cancer usually includes cisplatin [1]. Cisplatin causes tubular damage and acute kidney injury (AKI) [10, 11]. Although AKI is a long-term risk factor for chronic kidney disease and death [12, 13], no clinical studies have examined the association between cisplatin-induced AKI (c-AKI) and postoperative complications. We hypothesized that c-AKI during chemotherapy is associated with postoperative complications and investigated this in a retrospective cohort study.

## Methods

### Setting and patients

In this single-center, retrospective observational study, we included adult patients (age ≥ 18 years old) with no significant physical function problems (performance status ≤ 2) who had received planned preoperative chemotherapy including cisplatin and underwent transthoracic esophagectomy for esophageal cancer under general anesthesia between January 2017 and February 2022 at Nagoya City University Hospital. Patients who had underwent mediastinoscopic surgery, and colon reconstruction were excluded, as different surgical invasiveness could impact postoperative complications. We also excluded those with missing values including serum albumin, urinary protein, estimated glomerular filtration rate (eGFR), hemoglobin, and C-reactive protein (CRP) levels. The observation period ended when the patient was discharged from the hospital after surgery.

The study protocol was approved by the institutional review board at Nagoya City University Hospital (NO. 60-18-0008), and waiver of informed consent was approved by institutional review board (Nagoya City University Graduate School of Medical Sciences and Nagoya City University Hospital Institutional Review Board) due to the retrospective nature of the study.

### Operative procedure

All included patients underwent thoracoscopic esophagectomy, and intravenous methylprednisolone (Solu-Medrol 250 mg, Pfizer) was given immediately before surgery. Laparoscopy was used for abdominal manipulations, while patients undergoing pharyngo-laryngoesophagectomy and those with severe adhesions underwent open surgery.

### Exposure of interest and outcomes

The exposure of interest was c-AKI during preoperative chemotherapy. Outcomes were anastomotic leakage, postoperative pneumonia, and surgical site infection (SSI).

### Definitions

c-AKI was defined as stage 2 or higher AKI after preoperative chemotherapy within 10 days after chemotherapy using the KDIGO criteria (increase in serum creatinine to 2.0–2.9 times baseline, or reduction in urine output to < 0.5 mL/kg/h for ≥ 12 h) [14]. All patients were hospitalized for preoperative chemotherapy, and during hospitalization, urine output was measured every 6 h. When daily urine output was < 1500 mL or weight gain was more than 3 kg after chemotherapy, 20 mg furosemide was administered intravenously.

Anastomotic leakage was defined by imaging extraintestinal leakage on CT and fluoroscopy. Postoperative pneumonia was defined by the Uniform Pneumonia Score (van der Sluis et al. [15]) determined by body temperature, leucocyte count, and pulmonary radiographic findings. SSI was defined according to the guidelines of the Centers for Disease Control and Prevention [16].

Cancer staging was defined by the Japanese Classification of Esophageal Cancer, 11th Edition [17, 18]. Prechemotherapy and preoperative laboratory data were defined as those within 10 days before chemotherapy or surgery, and the closest to the date of chemotherapy or surgery, respectively. eGFR was calculated using the equation developed for the Japanese population by the Japanese Society of Nephrology [19].

### Statistical analyses

Data were presented as numbers (%) or medians (interquartile range). Demographic information for those with and without c-AKI was compared by Fisher’s exact test, the Mann–Whitney U test, or Wilcoxon signed-rank test. The length of hospital stay was compared between patients with and without c-AKI using Kaplan–Meier curves and log-rank tests. The trajectories of postoperative weight change, CRP levels, and eGFR were analyzed using a mixed-effects model, with time-dependent weight change, CRP, and eGFR levels as dependent variables and an interaction term between the cubic term of time and c-AKI as the independent variable. We included data up to the time before the event of interest or 20 postoperative days.

The associations between c-AKI and postoperative complications (anastomotic leakage, postoperative pneumonia, and SSI) were analyzed using logistic regression models. The data were adjusted for the logic of the propensity score (PS) for c-AKI. The PS was derived from models including age, sex, body mass index, eGFR, hemoglobin, CRP, albumin, urinary protein, Brinkman Index, height-adjusted total kidney volume measured on CT, type of neoadjuvant chemotherapy, pharyngo-laryngoesophagectomy, comorbidities and medications as listed in Table 1. These clinical variables used to create the propensity score were selected from the factors that have been reported as risk factors for postoperative complications and AKI [3–5, 10, 11]. Further adjustments for time-averaged eGFR, albumin levels, body weight ratio, and CRP were performed. Time-averaged values for these variables were defined as the average of these variables before the event of interest or within 20 days postoperatively. Sensitivity analyses were performed with inverse probability weighting (IPW). Data with IPW values at less than the 5th percentile or more than the 95th percentile were excluded from the analyses.

**Table 1:** Demographics

	Without c-AKI n = 79	With c-AKI n = 22	P-value
Age	72.0 (38.0–82.0)	70.5 (55.0–79.0)	0.70
Male sex	62 (78.8)	21 (95.8)	0.11
Body mass index (kg/m^2^)	20.3 (15.1–29.3)	20.2 (17.0–29.0)	0.64
eGFR (mL/min/1.73 m^2^)	74.7 (42.0–112.3)	72.1 (38.2–119.9)	0.87
Serum creatinine (mg/dL)	0.78 (0.63–0.89)	0.72 (0.64–0.87)	0.97
Performance status			0.82
0	69 (87.3)	19 (86.3)	
1	8 (10.1)	3 (13.6)	
2	2 (2.5)	0 (0)	
Hemoglobin (g/dL)	13.3 (9.3–17.2)	13.9 (10.9–15.8)	0.16
C-reactive protein (mg/dL)	0.14 (0.03–6.77)	0.20 (0.03–2.88)	0.86
Albumin (g/dL)	3.9 (2.7–4.9)	3.9 (2.9–4.6)	0.36
Urinary protein (+) or more	6 (7.6)	2 (9.1)	1.00
Brinkman Index	700 (0–85,260)	820 (0–2400)	0.70
HtTKV (mL/m^2^)	104.3 (76.4–160.6)	109.1 (73.5–151.5)	0.75
Preoperative chemotherapy; DCF/FP	45/34	16/6	0.22
Pharyngo-laryngoesophagectomy	6 (7.6)	2 (9.1)	1.00
Stage of esophageal cancer			0.86
0	2 (2.5)	0 (0)	
1	7 (8.9)	2 (9.1)	
2	25 (31.6)	6 (27.3)	
3	37 (46.8)	10 (45.5)	
4	8 (10.1)	4 (18.2)	
History of cerebrovascular accidents	2 (2.5)	1 (4.5)	0.53
History of diabetes mellitus	7 (8.9)	2 (9.1)	1.00
History of heart failure	1 (1.3)	1 (4.5)	0.39
History of hypertension	35 (44.3)	8 (36.4)	0.63
History of COPD	18 (22.8)	8 (36.4)	0.27
ACE inhibitors	0 (0)	0 (0)	NA
Antiplatelet agents	6 (7.6)	4 (18.2)	0.22
ARBs	21 (26.6)	6 (27.3)	1.00
Diuretics	1 (1.3)	0 (0)	1.00
Insulin	0 (0)	0 (0)	NA
NSAIDs	0 (0)	0 (0)	NA
SGLT2 inhibitors	1 (1.3)	0 (0)	1.00
Other anti-diabetic medications	5 (6.3)	2 (9.1)	0.64
Statins	5 (6.3)	4 (18.2)	0.10

Data were collected before chemotherapy. Data were shown as number (%) or median (interquartile range) as appropriate. *P*-values were calculated by Fisher’s exact test or Mann–Whitney U test

*c-AKI* cisplatin-induced acute kidney injury, *eGFR* estimated glomerular filtration rate, *HtTKV* height-adjusted total kidney volume (total kidney volume divided by height square), *DCF* Docetaxel + Cisplatin + 5 fluorouracil, *FP* Cisplatin + 5 fluorouracil, *COPD* chronic obstructive pulmonary disease, *ACE* angiotensin-converting enzymes, *ARB* angiotensin receptor blockers, *NSAIDs* non-steroidal anti-inflammatory drugs, *SGLT2* sodium-glucose cotransporters 2

We used mediation analysis to examine the indirect effect mediating the relationship between c-AKI and postoperative complications. The indirect effect is calculated as a comparison between the total effect of the exposure (AKI) and the effect of the exposure adjusted for intermediate variables. For the calculation of the mediation percentage, the indirect effect was then divided by the total effect of the outcome [20].

Mediation percentage was defined as;

$${\text{Mediation percentage}} = {{\left[ {100*\left( {{\text{OR}}1 - {\text{OR}}2} \right)} \right]} \mathord{\left/ {\vphantom {{\left[ {100*\left( {{\text{OR}}1 - {\text{OR}}2} \right)} \right]} {\left( {{\text{OR}}1 - 1} \right)}}} \right. \kern-0pt} {\left( {{\text{OR}}1 - 1} \right)}},$$

where OR1: odds ratio of c-AKI for anastomotic leakage (data adjusted for the logit of PS), OR2: odds ratio of c-AKI for anastomotic leakage (data adjusted for the logit of PS and covariates of interest).

A 1000 bootstrap resampling procedure was used to estimate the 95% confidence intervals (95%CI).

P-values < 0.05 were considered statistically significant. The incidence of anastomotic leakage and postoperative pneumonia after esophageal cancer surgery has been reported to be about 10% [2], and the incidence of cisplatin-induced AKI was about 30% [10]. We considered that it would be a clinically meaningful difference if 33% (one-third) of patients with cAKI develop these postoperative complications. A sample size of 101 gave a post hoc power of 0.73 at a significance level of 0.05.

Statistical analyses were performed using STATA MP v.17.1 (Stata Corp., College Station, TX).

## Results

**Fig. 1:**
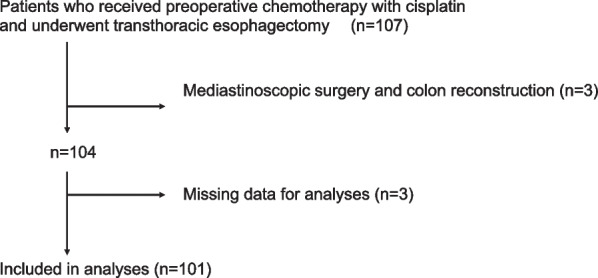
Flow of patients

During the study period, 107 subjects received preoperative chemotherapy with cisplatin and underwent transthoracic esophagectomy at Nagoya City University Hospital. After applying the exclusion criteria, data from 101 subjects were available for analysis (Fig. 1).

Among the 101 patients, 22 (21.8%) developed stage 2 or higher AKI. All patients with AKI met the diagnostic criteria by decreased urine output, while only one patient met the criteria by elevated serum creatinine. All patients were diagnosed with AKI before surgery [median (range) period from AKI diagnosis to surgery was 31 (16–59) days]*.* There was no patient with AKI due to sepsis or use of contrast media within 72 h before the onset of AKI.

Demographics were not significantly different between patients with and without c-AKI (Table 1).

**Fig. 2:**
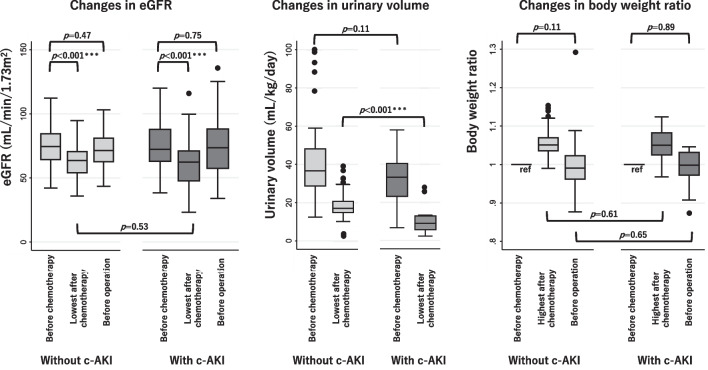
Changes in eGFR, urinary volume, and body weight for those with and without c-AKI. *eGFR* estimated glomerular filtration rate, *c-AKI* cisplatin-induced acute kidney injury. P-values were calculated by Mann–Whitney U test or Wilcoxon signed-rank test as appropriate. *** denotes statistical significance after Bonferroni correction

**Fig. 3:**
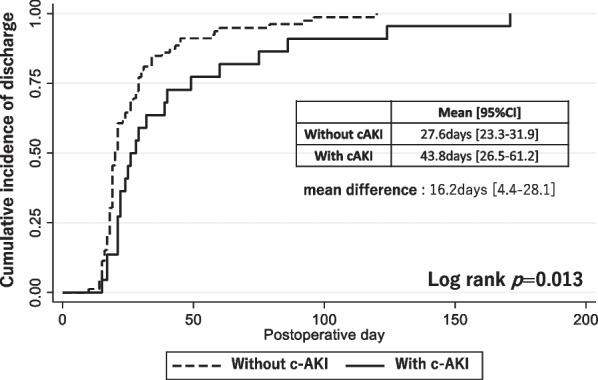
Cumulative incidence of hospital discharge for those with and without c-AKI. *c-AKI* cisplatin-induced acute kidney injury

Changes in eGFR, urinary volume and body weight among those with and without c-AKI are shown in Fig. 2. The lowest urine volume after chemotherapy was significantly lower in those with c-AKI. The lowest eGFR was significantly lower after chemotherapy than before chemotherapy for those with and without c-AKI, even after adjusting for multiple comparisons via the Bonferroni correction. However, there was no significant difference between those with and without c-AKI in the lowest eGFR, preoperative eGFR, or preoperative body weight (Fig. 2). Despite no significant difference in demographics and eGFR trajectories before surgery, the length of hospital stay for surgery was significantly longer for patients with c-AKI than for those without c-AKI [mean difference (95%CI) 16.2 (4.4–28.1) days] (Fig. 3). The duration of operation and intraoperative bleeding between patients with and without c-AKI were not significantly different [operation duration; 561 (527–683) min, 573 (519–632) min, P = 0.99, and intraoperative bleeding; 101 (74–207) mL, 121 (70–200) mL, P = 0.86, respectively].

**Fig. 4:**
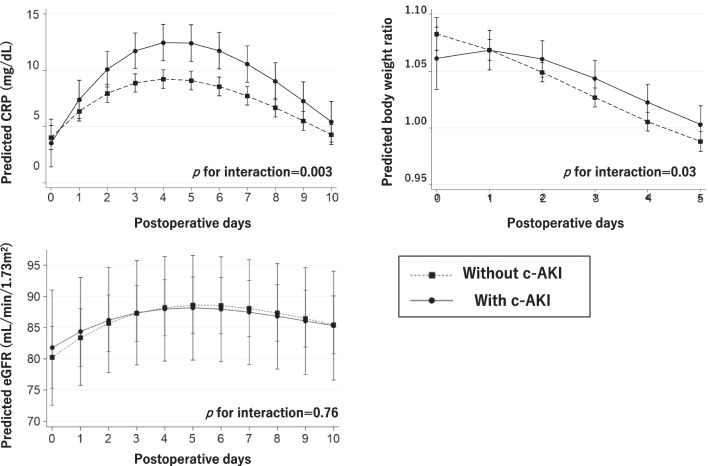
The trajectories of postoperative CRP, body weight ratio (postoperative body weight/preoperative body weight), and eGFR. The data were analyzed by the mixed-effects models with time-dependent CRP levels, body weight ratio, or eGFR as a dependent variable and including an interaction term between the cubic term of time and c-AKI as an independent variable. *CRP* C-reactive protein, *eGFR* estimated glomerular filtration rate, *c-AKI* cisplatin-induced acute kidney injury

Compared with those without c-AKI, patients with c-AKI had significantly higher CRP levels and body weight ratio (BW ratio) over time after surgery, whereas time-dependent eGFR levels did not differ between the two groups (Fig. 4).

Compared with those without c-AKI, the incidence of anastomotic leakage and postoperative pneumonia was significantly higher among those with c-AKI, whereas the incidence of SSI was not significantly different (Table 2). c-AKI was significantly associated with anastomotic leakage and postoperative pneumonia (Table 3). The association between c-AKI and anastomotic leakage did not change substantially after adjustment for the logit of the PS, or when analyzed by IPW. Postoperative pneumonia was no longer significantly associated with c-AKI after adjustment for the logit of the PS. However, the odds ratio remained greater than 2, suggesting a potentially important relationship. c-AKI was not associated with SSI in any of the analyses. The association between c-AKI and anastomotic leakage was attenuated by adjustment for time-averaged eGFR, albumin, body weight change, and CRP levels after surgery and before the event of interest, suggesting that these variables were potential mediators of the association. The association between c-AKI and postoperative pneumonia was also attenuated by adjustment for time-averaged albumin and CRP levels. The association between c-AKI and SSI was not affected by adjustment for these variables (Table 3).

**Table 2:** The incidence of postoperative complications within 20 days after surgery

	Without c-AKI n = 79	With c-AKI n = 22	P-value
Anastomotic leakage	8 (10.1)	8 (36.4)	0.01
Postoperative pneumonia	12 (15.2)	11 (50.0)	0.003
Surgical site infection	13 (16.5)	4 (18.2)	1.00

Data were shown as numbers (%). *P*-value was calculated by Fisher’s exact test

*c-AKI* cisplatin-induced acute kidney injury

**Table 3:** Association between c-AKI and postoperative complications

	Anastomotic leakage OR (95% CI)	Postoperative pneumonia OR (95% CI)	Surgical site infection OR (95% CI)
Univariate analysis	4.14 (1.30–13.18)	3.87 (1.35–11.0)	0.80 (0.21–3.11)
PS adjustment*	5.78 (1.50–22.12)	3.87 (1.35–11.03)	1.71 (0.38–7.70)
Inverse probability weighting^#^	5.77 (1.42–23.51)	2.23 (0.67–7.33)	2.00 (0.02–1.72)
PS adjustment with stepwise adjustment for potential mediators			
Model 1; PS adjustment*	5.78 (1.50–22.12)	3.87 (1.35–11.03)	1.71 (0.38–7.70)
Model 2	5.00 (1.27–19.66)	2.27 (0.67–7.72)	1.90 (0.41–8.71)
Model 3	4.28 (1.06–17.31)	1.77 (0.50–6.32)	1.77 (0.38–8.33)
Model 4	3.86 (0.92–16.11)	2.17 (0.57–8.26)	1.61 (0.34–7.77)
Model 5	2.45 (0.50–11.95)	1.84 (0.48–7.12)	1.80 (0.36–8.99)

PS derived from models including age, sex, body mass index, estimated glomerular filtration rate, hemoglobin, C-reactive protein, albumin, urinary protein, Brinkman Index, chronic obstructive pulmonary disease, height-adjusted total kidney volume, kinds of preoperative chemotherapy, pharyngo-laryngoesophagectomy, comorbidities, and medications listed in Table 1

Model 1: The data were adjusted for the logit of propensity score

Model 2: adjusted for variables in Model 1 and time-averaged estimated glomerular filtration rate before the event of interest or within 20 days postoperatively

Model 3: adjusted for variables in Model 2 and time-averaged albumin levels before the event of interest or within 20 days postoperatively

Model 4: adjusted for variables in Model 3 and time-averaged body weight ratio (postoperative body weight/preoperative body weight) before the event of interest or within 20 days postoperatively

Model 5: adjusted for variables in Model 4 and time-averaged C-reactive protein (log-transformed) levels before the event of interest or within 20 days postoperatively

*c-AKI* cisplatin-induced acute kidney injury, *PS* Propensity score

*Data were adjusted for the logit of the propensity score

^#^Data with inverse probability weighting values less than 5 percentile or more than 95 percentiles were excluded, which left 89 cases in the analyses

**Fig. 5:**
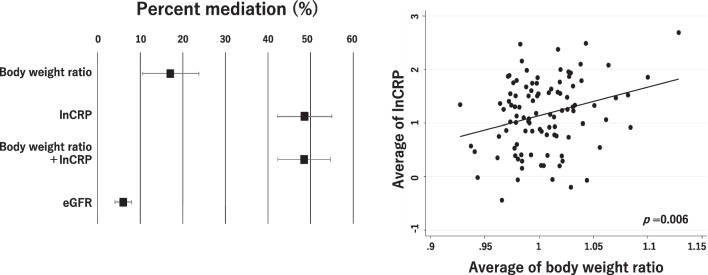
Mediation by body weight ratio (postoperative body weight/preoperative body weight), C-reactive protein, or eGFR for the association between c-AKI and anastomotic leakage. Scatter plots for the average C-reactive protein (natural log-transformed) level and the average body weight ratio. The regression line was shown in the scatter plot. Mediation percentage was defined as [100*(OR1–OR2)]/(OR–1). OR1: odds ratio of c-AKI for anastomotic leakage (Data adjusted for the logit of propensity score). OR2: odds ratio of c-AKI for anastomotic leakage (Data adjusted for the logit of propensity score and covariates indicated in the graph). *c-AKI* cisplatin-induced acute kidney injury, *lnCRP* C-reactive protein (natural log-transformed), *CRP* C-reactive protein, *eGFR* estimated glomerular filtration rate

Mediation analysis showed that body weight changes and CRP levels mediated the association between c-AKI and anastomotic leakage by 17% and 48%, respectively. The mediation percent did not change when the model include both CRP and body weight change. Scatter plots for an average of CRP and BW ratio show a significant association between CRP and BW ratio (Fig. 5).

## Discussion

This study showed that c-AKI diagnosed only by urine output criteria after chemotherapy was associated with postoperative complications, especially anastomotic leakage, leading to a significantly longer hospital stay. The association was mainly mediated by higher CRP after surgery.

This is the first study to show that c-AKI during preoperative chemotherapy was associated with postoperative complications. As shown in Table 1, there were no significant differences in known risk factors for postoperative complications between patients with and without c-AKI. After adjusting for these known risk factors, c-AKI remained independently associated with anastomotic leakage. From these two viewpoints, c-AKI is a novel risk factor for postoperative complications in patients with esophageal cancer. Numerous observational studies have shown that AKI was independently associated with worse prognoses including all-cause mortality, cardiovascular morbidity, and end-stage kidney disease [12, 21, 22]. However, baseline characteristics in these studies were significantly different between those with and without AKI. As a result, the association between AKI and worse prognosis could be due to residual confounders. Our study was peculiar because the background of those with and without c-AKI was not significantly different. Therefore, our results strengthened the argument for an independent association between c-AKI and postoperative complications.

Our study was also unique since most patients with c-AKI were diagnosed only by urine output criteria, which could be overlooked in real clinical practice. An evaluation of kidney function using serum creatinine level may be insufficient. Patients with c-AKI may have undergone surgery before the complete recovery of their tubular function. It would have been beneficial to measure clinical biomarkers for tubular damage, which might allow us to infer an appropriate surgical timing. Otherwise, preoperative chemotherapy with cisplatin might have acted as a stress test for the kidneys to evaluate the poor functional reserve that cannot be determined by existing renal evaluations. A stricter in–out balance may be required during perioperative management for patients with c-AKI. Therefore, the clinical application of biomarkers as indicators of perioperative fluid delivery and balance is also required [23, 24].

The underlying mechanism of the association between c-AKI and postoperative complications could be prolonged inflammation after AKI. We also demonstrated that the time-averaged BW ratio was associated with time-averaged CRP level after surgery, suggesting that those with prolonged elevated CRP levels had prolonged fluid retention. In recent years, kidney tubules have been reported to play a role in the suppression of inflammation [25, 26]. For example, kidney injury molecule 1 expression is anti-inflammatory due to its mediation of the phagocytotic process in tubules [27]. Additionally, in a clinical study, prolonged inflammation was suggested to be a mediator of worse outcomes after AKI [28]. The results of the current study suggest that the kidneys after c-AKI did not suppress the postoperative inflammatory response and that increased vascular permeability due to proinflammatory cytokines caused prolonged weight gain with systemic or local edema, leading to anastomosis leakage and postoperative pneumonia. Niebauer et al. reported that in patients with edema due to congestion, intestinal edema causes increased intestinal permeability, leading to bacterial translocation, which, in turn, leads to a prolonged inflammatory response and more severe edema, resulting in a vicious cycle. If a similar mechanism is observed in patients with c-AKI, diuretic use with body weight as an indication may help reduce the occurrence of postoperative complications [29].

This study has several limitations including its retrospective design and the small number of patients at a single center. Although the sample size was relatively small, our results showed that the incidence of anastomotic leakage among patients with and without cAKI and the incidence of cAKI were 10.1%, 36.4%, and 21.8%, respectively, which were not substantially deviated from our assumptions for power calculation. Due to the small number of patients, we were unable to examine the relationship between AKI severity (AKI stage) after preoperative chemotherapy and postoperative complications.

## Conclusion

Cisplatin-induced AKI after preoperative chemotherapy in esophageal cancer patients was significantly associated with the development of postoperative complications and led to a resultant longer hospital stay. This association was mainly mediated by higher CRP level in those with c-AKI. Increased vascular permeability and tissue edema might explain the mechanisms for the higher incidence of postoperative complications. Conservative fluid management and liberal use of diuretics or albumin might be warranted for those undergoing surgery for esophageal cancer with a history of c-AKI.

## Data Availability

The datasets used and analyzed during the current study are available from the corresponding author on reasonable requests.

## Abbreviations

AKI: Acute kidney injury
c-AKI: Cisplatin-induced acute kidney injury
eGFR: Estimated glomerular filtration rate
95%CI: 95% Confidence interval
CRP: C-reactive protein
SSI: Surgical site infection
PS: Propensity score
IPW: Inverse probability weighting
BW ratio: Body weight ratio
